# Muscle Selection for Focal Limb Dystonia

**DOI:** 10.3390/toxins10010020

**Published:** 2017-12-29

**Authors:** Barbara Illowsky Karp, Katharine Alter

**Affiliations:** 1 Combined NeuroScience IRB, National Institute of Neurological Disorders and Stroke, National Institutes of Health, Bethesda, MD 20892, USA; 2 Department of Rehabilitation Medicine, National Institutes of Health, Bethesda, MD 20892, USA; kalter@cc.nih.gov

**Keywords:** limb dystonia, writer’s cramp’ musician’s cramp, lower limb dystonia, runners dystonia, chemodenervation, botulinum toxin, muscle selection, gait testing

## Abstract

Selection of muscles for botulinum toxin injection for limb dystonia is particularly challenging. Limb dystonias vary more widely in the pattern of dystonic movement and involved muscles than cervical dystonia or blepharospasm. The large variation in how healthy individuals perform skilled hand movements, the large number of muscles in the hand and forearm, and the presence of compensatory actions in patients with dystonia add to the complexity of choosing muscles for injection. In this article, we discuss approaches to selecting upper and lower extremity muscles for chemodenervation treatment of limb dystonia.

## 1. Introduction

### 1.1. Background, Genetics, Pathophysiology: Focal Upper Limb Dystonia

The current definition of dystonia describes it as “a movement disorder characterized by sustained or intermittent muscle contractions causing abnormal, often repetitive, movements, postures, or both. Dystonic movements are typically patterned, twisting, and may be tremulous. Dystonia is often initiated or worsened by voluntary action and associated with overflow muscle activation [1]”. These features are exemplified by focal hand dystonia, especially those that are task-specific. Axis I of the 2013 revised consensus criteria for dystonia includes five descriptors: age at onset, body distribution, temporal pattern, and coexistence of other movement disorders and other neurological manifestations [1]. Within this nosology, focal hand dystonia is a condition of early-late adulthood; the usual age of onset is in the 4th–5th decade [2,3]. Limb dystonia, is “focal” when limited to a single limb or part of a single limb. However, the limbs are often involved in hemi-, segmental, multifocal, and generalized dystonias. Even in patients with writer’s cramp, the proximal arm muscles including the shoulder girdle may be involved and symptoms can be bifocal, affecting both hands. Adult onset focal limb dystonia frequently evolves to its full expression over weeks to months. Once fully manifest, adult onset limb dystonia tends to be static but can progress, even years later. Spread tends to be towards proximal muscles and to the contralateral limb [4,5,6]. Tremor, similar in nature to essential tremor, is a not uncommon accompaniment to limb dystonia [7].

Regarding axis II of the current dystonia classification schema, which characterizes dystonias based on etiology, the limbs are involved in many symptomatic dystonias including those associated with structural nervous system pathology. Many focal limb dystonias, unassociated with other neurologic signs, are currently considered sporadic, although as genes associated with dystonia continue to be identified, they are likely to be reclassified as inherited. About 25% of those with focal hand dystonia have a family history of dystonia [8,9,10]. Known genetic disorders can be identified in some patients with apparently focal limb dystonia, such as dopa-responsive dystonia, DYT6 and others [11,12]. An association has also been reported for variants in the gene for arylsulfatase G with the focal task-specific dystonias writer’s cramp (WC) and musician’s cramp (MC) [13,14].

The pathophysiology of WC and MC has been particularly well-studied, as the limb in task-specific dystonia can be evaluated without activation of the dystonia, allowing comparison between dystonic and non-dystonic actions. In addition, the affected hand can be compared to the unaffected hand. Studies in WC and MC have shown a loss of inhibition at multiple levels and enhanced cortical plasticity [15,16,17]. Since many of these changes can be found bilaterally, they are trait states and do not fully explain the manifestation of dystonia.

Focal limb dystonia of the upper or lower extremity is often task-specific, present only during the performance of a particular activity. In task-specific dystonia, other activities, including those using the same muscles, are performed normally. In task-specific focal limb dystonia, the muscles affected typically encompass those used during a repetitive activity. Thus, writer’s cramp is the most common focal hand dystonia, initially reported in the 18th century as “scrivener’s palsy” [18]. Similar symptoms in other professions led to the term “occupational palsies”, affecting tailors, cobblers, musicians and others performing skilled, usually fine-motor, repetitive tasks [7,19].

Estimates of the prevalence of focal hand dystonia range from 7–15/100,000 population [20,21]. Musician’s dystonia is thought to affect up to 1% of professional musicians [22]. Unlike most other adult onset focal dystonias that typically affect women, writer’s cramp affects men and women about equally, while musician’s dystonia is more prevalent in men [23]. Focal hand dystonia is associated with a diminished quality of life and can end careers of professional musicians [24,25]. Over-practice or overuse likely contributes to the emergence of dystonic symptoms, which may be a manifestation of a genetic predisposition. Personality factors may also contribute to its expression. In particular, musician’s dystonia has been associated with more anxiety, stress and perfectionism compared to musicians without dystonia [26].

### 1.2. Treatment of Focal Hand Dystonia

Even before seeking medical attention, many patients with focal hand dystonia try to alter their performance of affected tasks, equipment or instruments. Those with writer’s cramp try altering their grip on the pen or new, often thicker, pens. Musicians try to adjust their technique or instrument. These approaches rarely provide more than minimal to mild benefit. Oral medications are similarly ineffective. Given the lack of effective alternatives, botulinum toxin has become the treatment of choice for focal hand dystonia [27,28].

### 1.3. Botulinum Toxin for Focal Hand Dystonia

There are many aspects to consider when approaching injection with botulinum toxin including selection of toxin type and brand, toxin dose and dilution. There are currently four marketed brands of toxin in the US, abotulinumtoxinA (Dysport^®^, IPSEN, Basking Ridge, NJ, USA), incobotulinumtoxinA (Xeomin^®^, Merz North American Headquarters, Raleigh, NC, USA), onabotulinumtoxinA (Botox^®^, Allergan Inc., Irvine, CA, USA) and rimabotulinumtoxinB (Myobloc^®^, Solstice Neurosciences, LLC, Louisville, KY, USA). None are approved by the U.S. Food and Drug Administration (FDA) for the treatment of focal hand dystonia (FHD) and there is little information available that informs the choice of toxin. Thus, toxin selection is frequently predicated on practitioner preference and familiarity, cost and convenience. Similarly, while there are dosing guidelines for the toxins approved for upper limb spasticity, there is little consensus information on dosing for focal hand dystonia, although the FHD doses are typically lower than those used to treat spasticity in the same muscles. The mean starting dose in one retrospective series of botulinum toxin (BoNT) for FHD was 25 units of onabotulinumtoxinA; optimal effective dose among patients continuing injections for at least 10 years was 50 units [3,29]. A retrospective series of 56 musicians treated for a mean 23 months reported a mean starting dose of abobotulinumtoxinA of 127 units and final dose of 112 units [29]. In general, dosing starts at the low end of the potentially therapeutic range with titration over successive injection sessions until benefit is maximized and weakness or other adverse effects are minimized [3]. The total dosing is, importantly, based at least in part on the particular muscles to be injected and the number of muscles to be included in a given session.

## 2. Muscle Selection for Upper Limb Botulinum Toxin Injection

A key component of the key to success of botulinum toxin therapy for dystonia is appropriate selection of muscles for injection. Muscle selection in FHD is not straightforward: the forearm alone has approximately 20 muscles, including some muscles where separate component fascicles mediate individual digit actions. In addition, one or more than one muscle may contribute to the same movement or action. Table 1 delineates major muscles contributing to different acting on elbow, wrist and finger joints (Table 1). 

Hand movement is intricate and tightly coordinated. Thus, the muscle interactions underlying fine hand motor functions are complex; the distortion of muscle interaction in dystonia adds to that complexity. The identification of which muscles are involved in dystonic movements is further complicated by the presence of compensatory muscle activity, which is often subconscious. Many patients have difficulty identifying which of their muscles are actively dystonic and which are involuntarily trying to counteract the dystonia. Muscle discomfort is similarly not a straightforward indication of dystonia, as it can also arise both from excessive dystonic contraction and anti-dystonic compensation. 

The first line of approach to muscle selection is a careful history. The pattern of abnormal contraction at the onset of symptoms should be discussed and may be a clue to the primary dystonic involvement. The patient should be questioned about how and which activities are solely or disproportionately affected and to report where they feel muscle tension. They should be asked about their perception of which areas are involved and direction of movement distortion. 

**Figure 1:**
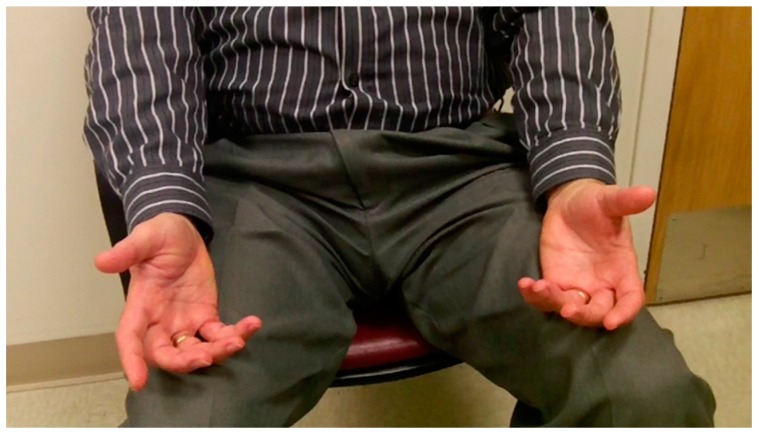
Hand dystonia at rest: posturing of both hands.

The next line of approach is physical examination. A careful neurologic examination should be done at least on initial evaluation to rule out non-dystonic conditions as a cause of the symptomatology. Abnormal sensory loss, reflex changes or weakness would indicate a non-dystonic disorder. The examination can then focus on the dystonia. The key to being able to interpret the findings on examination is a knowledge of functional anatomy, including which muscles cross each joint and can contribute to the pattern of posture and movement observed [30]. The patient should be examined initially at rest as, when FHD is severe, abnormal posture is present at rest (Figure 1). There may be decreased arm swing or dystonic posturing on walking.

**Figure 2:**
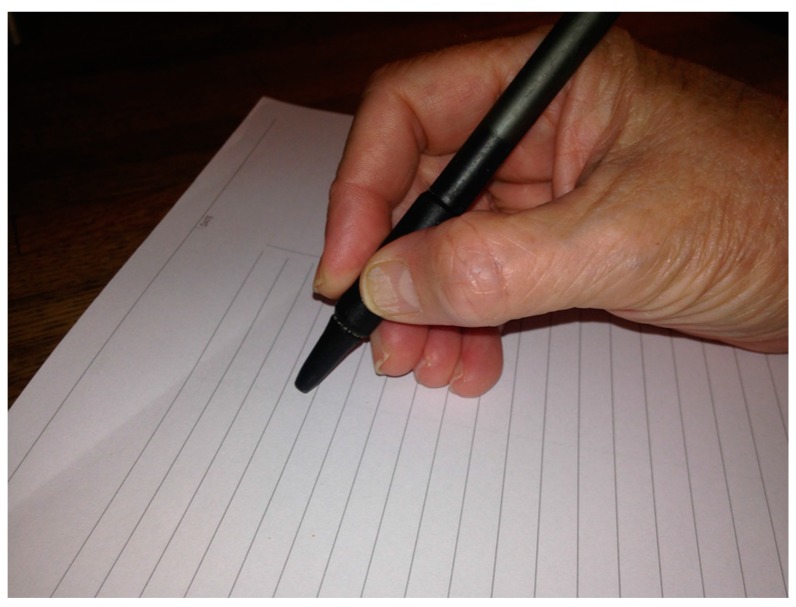
Typical tripod pen grip in a right-handed writer.

Next, the examination should focus on the activities provoking the dystonia. The patient should first be asked to perform the activities as normally as possible and then again using any adaptations or compensatory actions employed. During the examination with actions, the patients should be asked to point out anatomic areas of tension and to describe how the dystonia alters their intended posture or performance. Throughout these evaluations, the practitioner should have an understanding of what the action looks like when performed by unaffected individuals and the patient’s intended pattern. For example, a typical tripod pen grip has the fingers flexed at the metacarpal and interphalangeal joints (Figure 2).

In a right-handed individual, the wrist tends to be slightly flexed when starting a sentence on the left side of a piece of paper and extends as the pen moves across the paper to the right. In left-handed writers, the wrist may be more flexed so that the writing is not smeared as the pen moves from left to right. However, in both left- and right-handers, the position of wrist and individual fingers is highly variable during writing. To fully visualize the posture of all fingers during writing when evaluating writer’s cramp, the writing hand should be observed from both dorsal and palmar aspects. Additional observation from above the writing surface will enable better visualization of wrist flexion or extension. Inspection from the front allows identification of proximal arm and limb girdle position. It is important to check for forearm pronation and supination as well as wrist and finger deviation.

During examination, muscle selection can be aided by the examiner applying pressure that counters the apparent dystonic movement. For example, in a patient where there is concern that pronation is contributing to dystonic disability, the examiner can apply pressure in the antagonist direction of supination while the patient writes or performs the dystonic-eliciting activity. Improvement with counter-pressure indicates likely involvement of the agonist muscle.

Whenever possible, musicians should be evaluated playing their instrument using music selected to provoke the dystonia. Musicians and others with occupational hand dystonia should be asked about their usual or intended upper limb position when playing as approaches to the instrument and technique vary widely. As well as appreciating what the intended or “normal” posture is for performing the affected activity, it is helpful for the practitioner to be knowledgeable about commonly affected muscles for different types of FHD. In writer’s cramp, flexion dystonia is more common than extension [31]. In musicians, the intricacy demanded of the hand or fingers influences the dystonic pattern. In pianists, higher technical demand is usually on the right hand compared to the left, so dystonic flexion of the right 4th and 5th fingers is disproportionately seen. In violinists, the higher technical demand is on the fingers of the left hand, which is hence more frequently affected by dystonia [32]. 

Close communication during examination is critical. Because a writing hand normally has flexed fingers, it can be difficult for an examiner to appreciate superimposed dystonic finger flexion. However, the patient may report a “death grip” on the pen. In musicians especially, careful discussion of hand and finger involvement can avoid misunderstanding. The neurology literature and pianists often number the fingers 1 through 5, starting with the thumb. However, violinists do not use the thumb of the left hand on the strings and therefore number their fingers 1 through 4, starting with the index finger. Thus, the index finger would be called digit “2” by a pianist but would be digit “1” for a violinist. 

**Figure 3:**
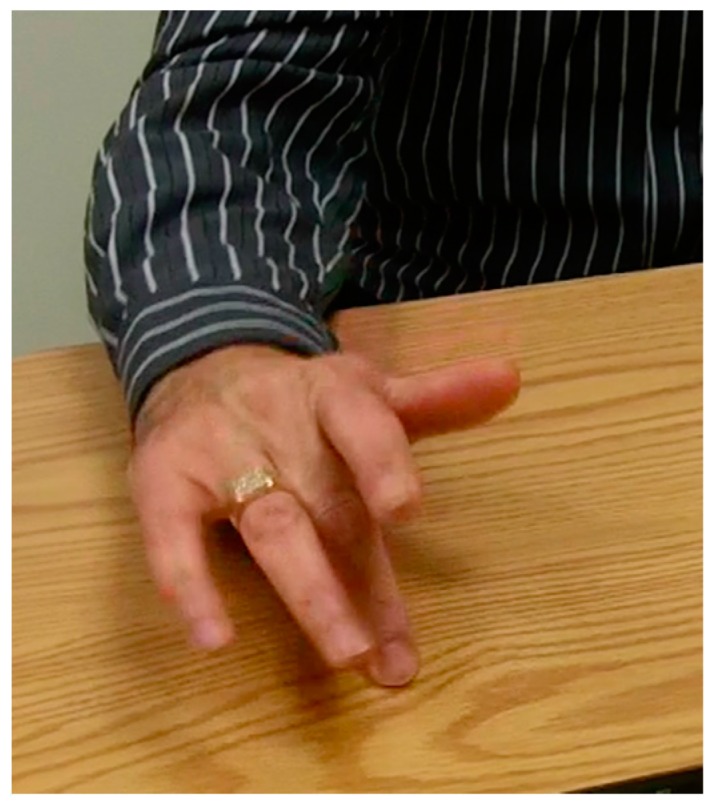
Dystonic finger extension on attempted sequential finger tapping.

Examination should also include observation of the performance of other activities that might elicit dystonia without confounds, such as holding a pen or playing an instrument. A simple five-finger tapping exercise can bring out dystonic posturing in some patients with FHD (Figure 3). 

**Figure 4:**
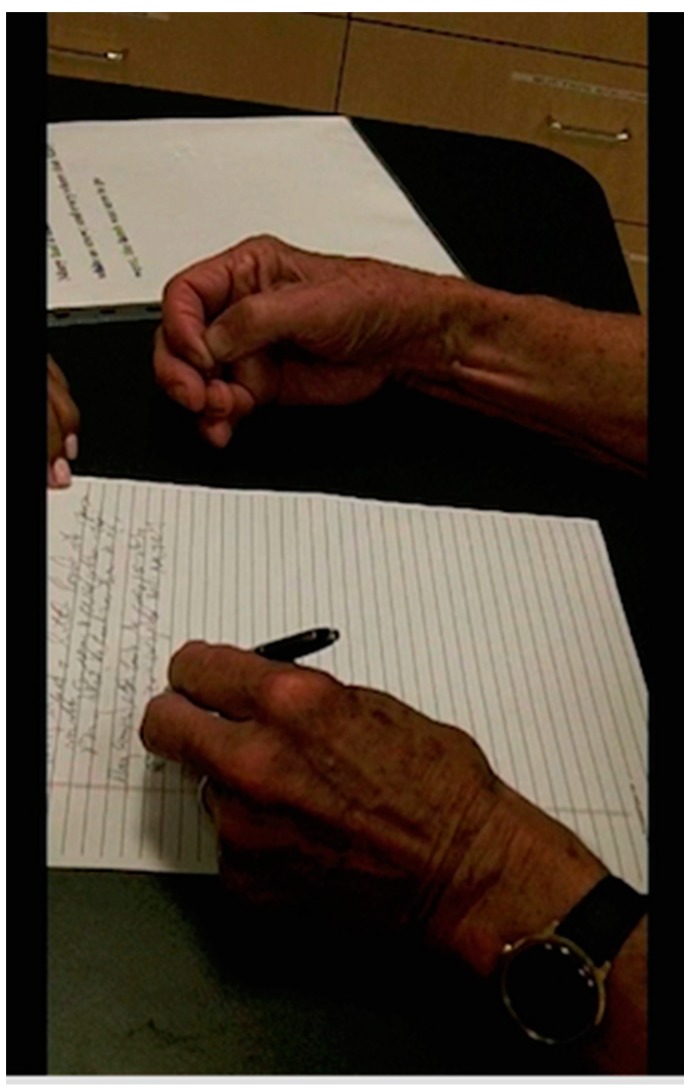
Mirror dystonia of the dominant hand when writing with a non-dominant hand.

Mirror movements, in which involuntary dystonic posturing of the affected limb is evoked by performance of tasks by the uninvolved contralateral limb, is present in about 50% of those with focal hand dystonia. When present, mirror movement can demonstrate involved muscles unmasked by the presence of compensatory movements [33,34,35], which can aid selection of muscles for injection (Figure 4). 

Injection often proceeds based on the history and physical examination and understanding of which muscles contribute to particular action at upper extremity joints. While it is important to evaluate carefully and document the likely involved muscles, it is not necessary to inject all muscles contributing to the abnormal posture. Injection of the primary muscles involved in the dystonia may be adequate to achieve significant benefit. Muscle selection is then refined over the course of subsequent injection sessions based on patient response and satisfaction.

Accurate targeting of upper extremity muscles is also the key to successful injection. The position and depth of muscles varies greatly in individuals and may change over time and with repeated injection. One also needs to consider normal anatomic variants, such as the presence or absence of palmaris longus. Needle placement based on anatomic landmarks has been shown to be inaccurate [36], especially when targeted to specific finger fascicles of compound muscles such as flexor digitorum superficialis or profundus. A localization technique to assure that the injection is given accurately into the selected muscle, such as electromyography (EMG), electrical stimulation or ultrasound, is recommended for upper extremity injections. 

### 2.1. Ancillary Techniques for Muscle Selection for Upper Limb Dystonia

When the response to injection is unsatisfactory or if physical examination alone seems insufficient to identify muscle involvement, ancillary testing may be helpful. 

### 2.2. Muscle Afferent Block with Lidocaine

Early reports on the pathophysiology of focal hand dystonia observed that dystonic symptoms could be elicited by vibration to the palm or flexor forearm tendons, presumably mediated by muscle afferents. The dystonic movements were alleviated by local injection of lidocaine for as long as 24 h, attributed to lidocaine’s blockade of alpha1 and gamma afferents. While intramuscular lidocaine can relieve dystonia [37,38,39,40], it is of limited therapeutic value for dystonia as the benefit is short-lasting. The quick onset (within 5–15 min) and short duration of action, however, make lidocaine potentially helpful in selecting muscles for injection. A muscle possibly involved in the dystonia and under consideration for botulinum toxin treatment can be injected with lidocaine at a dose that blocks muscle afferents but not alpha motor neurons. Improvement in dystonia over the hour following lidocaine installation implicates that muscle’s involvement in the dystonia and indicates its appropriateness for toxin injection. 

### 2.3. Surface and Fine Wire Electromyography (EMG)

**Figure 5:**
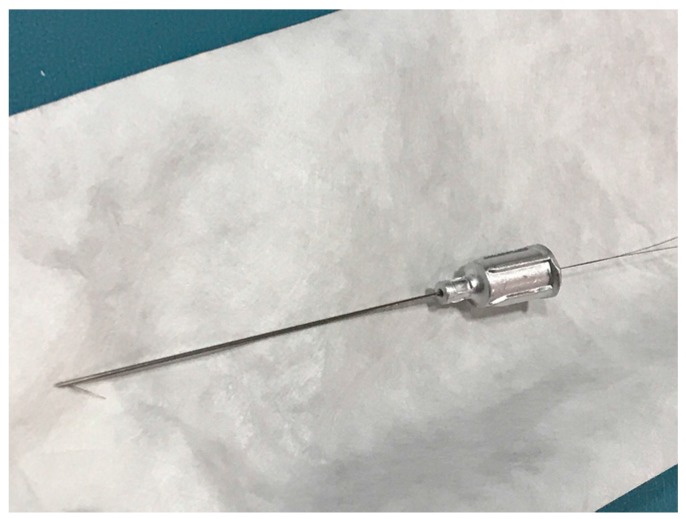
Needle threaded with hooked wires.

Dystonia is characterized electromyographically by prolonged contraction of muscles, co-contraction of agonist/antagonist pairs, and recruitment of muscles not normally involved in an activity [41]. EMG can therefore be helpful in understanding which muscles may be involved in the dystonia, although dystonic and compensatory actions may be difficult to differentiate by EMG. When deep muscles not amenable to direct visualization are implicated in dystonic activity, wire EMG may be of particular use [42]. In this technique, thin wires that are Teflon coated except for the barbed tip are threaded through a needle (Figure 5).

**Figure 6:**
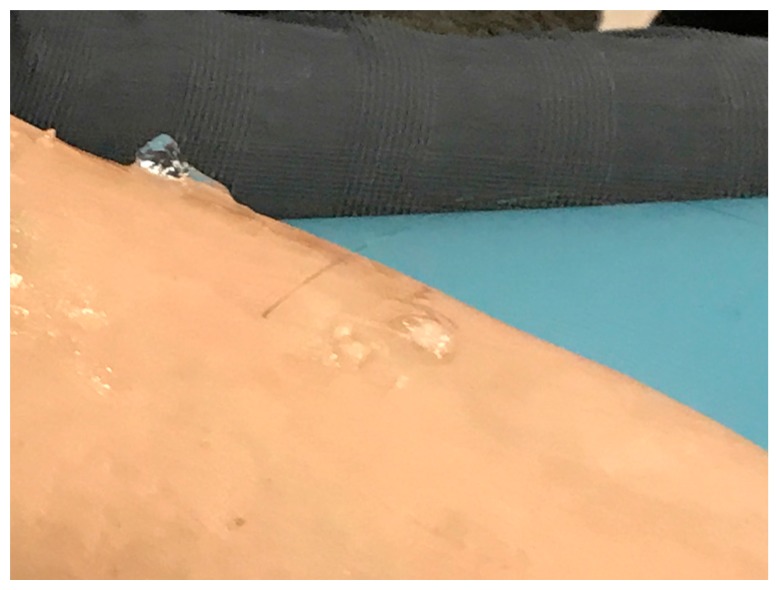
Wire electrode in place in the muscle.

The needle with wires is inserted into the muscle selected for study. The needle is then carefully withdrawn, leaving the wires embedded in the muscle (Figure 6).

Once in place, the wire is barely felt by the patient, who can then perform dystonic and non-dystonic eliciting activities while the EMG is recorded. With a multichannel machine, wires can be placed in several muscles, including agonist/antagonist pairs or groups of muscles and activity recorded simultaneously. Overactivity of a muscle or contraction with an activity, during which it is usually silent, suggests that the muscle is dystonic and should be injected. 

### 2.4. Imaging

Ultrasound or other muscle imaging may demonstrate hypertrophy in dystonic muscles. However, muscle imaging has not been shown to be helpful in selecting muscles for injection. 

## 3. Muscle Selection for Lower Limb Dystonia

The lower limb is commonly involved in many generalized dystonias including DYT1 and DYT5 (dopa-responsive dystonia (DRD)) as well as in dystonias secondary to other disorders, such as Parkinson’s Disease, stroke, or cerebral palsy. Adult-onset, focal lower limb dystonia is less common [43,44,45]. Focal leg dystonia is more common in women than men and typically of middle age onset [44]. Similar to upper limb dystonia, lower limb dystonia can be task-specific; for example, cases have been reported of leg dystonia only when walking down steps [46]. The most common task-specific focal lower extremity dystonia is “runner’s” dystonia [47]. 

As with other dystonias, lower limb dystonia may be initially task-specific, for example, present only during running. The task-specificity can be lost over time and other actions, including walking, can be affected. Dystonic postures in the leg frequently involve foot inversion, often with toe flexion or extension. There may also be knee and/or hip flexion or extension. Of note is that gait may improve or normalize on walking backwards.

### 3.1. Treatment of Lower Limb Dystonia

Because the leg is commonly affected in DRD and since leg dystonia can be a presenting symptom of Parkinson’s Disease, patients should receive a trial of l-dopa. Other medications, such as anticholinergics, often offer only minor benefit and may be associated with significant adverse effects at the doses required. Phenol neurolysis can be used in selected patients [48]. For most, however, the treatment of choice is botulinum toxin.

### 3.2. Muscle Selection for Lower Limb Botulinum Toxin Injection

As with injections for upper limb dystonia, appropriate selection of lower limb muscles for injection is mandatory for successful treatment. The medical history will provide information on possible preceding trauma. The patient should be asked to describe the movements and the actions that precipitate dystonic contraction. A history of diurnal fluctuation may suggest dopa-responsive dystonia. Physical examination can help to rule out Parkinsonian syndromes. The gait examination follows. The patient should be examined walking, both with and without shoes. The shoes can also be checked, as abnormal wear patterns of the sole and upper shoe may provide clues as to nature and direction of postural distortions. Stress gaits, such as walking on the toes or heels, may bring out additional dystonic posturing. Patients should be asked to walk backwards; normalization of gait on walking backwards demonstrates the task specificity of the dystonia. Runners should be observed while running in addition to walking. If the patient has sensory tricks or gestes antagonists that help, they should be displayed. 

In evaluating patients with lower extremity dystonia, the practitioner should have an understanding of the normal gait cycle and of functional anatomy so that abnormal patterns can be appreciated (Table 2).

### 3.3. Ancillary Techniques for Muscle Selection for Lower Limb Dystonia

**Figure 7:**
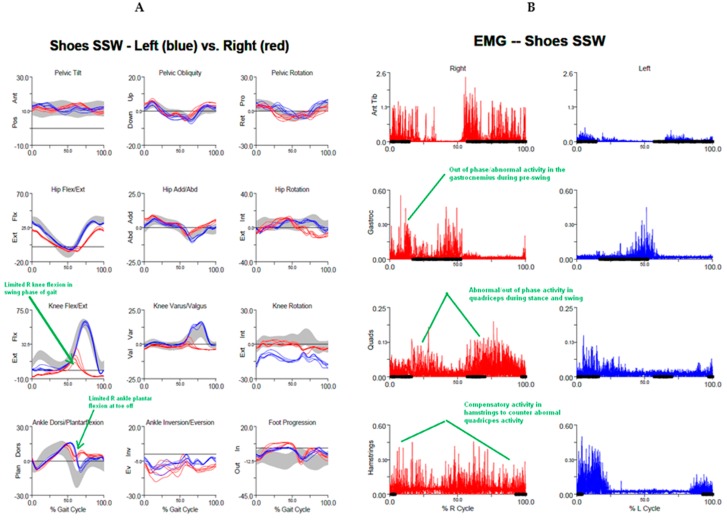

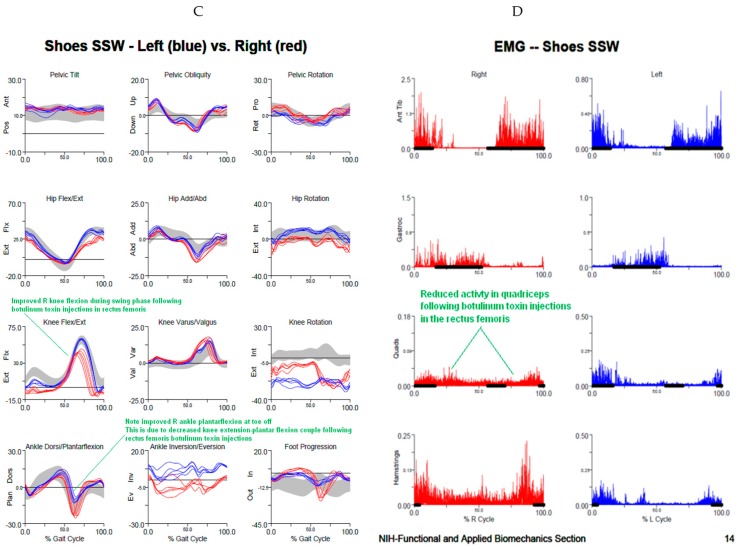
Gait analysis in patients with focal dystonia, before and after botulinum toxin injection. (**A**) self-selected pace walking before injection shows limited right knee flexion in swing phase of gait and limited right ankle plantar flexion at toe off; (**B**) self-selected pace walking before injection: Electromyography (EMG) shows abnormal out of phase activity in right gastrocnemius during pre-swing and in quadriceps during stance and swing, with abnormal compensatory activity in hamstrings; (**C**) self-selected pace walking after injection shows improved right knee and plantar flexion; (**D**) self-selected pace walking after injection: EMG shows reduced activity in quadriceps following injection of rectus femoris and in compensatory activity in hamstrings.

As with upper limb dystonia, lidocaine afferent block and surface and/or fine wire EMG may be helpful in identifying dystonic muscles in the lower limb. Walking is a stereotyped activity, with identifiable phases and established patterns of phasic coordination of muscle agonist and antagonist activation at each stage. Formal gait analysis includes monitoring gait kinematics and muscle activity by surface or wire EMG, and often with video motion capture during performance of various set walking tasks. A formal gait analysis may be helpful in characterizing how the pattern of muscle contraction in a patient with dystonia differs from standard gait norms, including identification of muscles contracting during phases of gait when they should be silent and co-contraction of agonist/antagonist pairs [49]. As well as part of muscle selection, motion analysis may be useful when repeated after treatment to assess gait response to injection (Figure 7).

## 4. Conclusions

Correct identification of muscles involved in dystonia and selection for injection is especially critical for successful outcome in treating upper and lower extremity dystonia with botulinum toxin injection. Selection of muscles is not straightforward in the upper extremity due to the complexity of forearm, hand and finger skilled movement and the number of muscles that can possibly contribute to those movements. Evaluation of lower extremity dystonia is aided by gait analysis and the availability of normative gait data. Careful physical examination and evaluation at rest, with activities directly evoking the dystonia, with mirror movements and with other tasks, are the mainstays of initial muscle selection. Observation of response to toxins over time allows the practitioner to adjust and refine muscle selection and doses to optimize botulinum toxin treatment for dystonia.

## Figures and Tables

**Table 1:** Muscles of upper extremity; primary muscles are in bold.

**Elbow**			
Flexion		Extension	
	**biceps**		**triceps**
	**brachialis**		anconeus
	**brachioradialis**		
	Pronator teres		
	ECR		
	FCR		
Pronation		Supination	
	**Pronator teres**		**supinator**
	**Pronator quadratus**		biceps
			
**Wrist**			
Flexion		Extension	
	**FCR**		**ECR**
	**FCU**		**ECU**
	FDS		EDC
	FDP		EIP
	Palmaris longus		EDM
	FPL		EPL
Radial deviation		Ulnar deviation	
	**FCR**		**FCU**
	**ECR**		**ECU**
**Fingers**			
MP flexion		extension	
	**FDS**		**EDC**
	FDP		**EDM**
	lumbricals		**EIP**
	interossei		lumbricals
			interossei
PIP flexion		PIP extension	
	**FDS**		**EDC**
	FDP		**EIP**
abduction		adduction	
	**Dorsal interossei**		**Palmar interosei**
	EDC		FDS
	EIP		FDS
	ADM		
**Thumb**			
flexion		Extension	
proximal		proximal	
	**FPB**		**EPB**
	OP		
	APB		
	FDI		
distal		distal	
	**FPL**		**EPL**
			**APL**
abduction		adduction	
	**APL**		**Add Pol**
	**APB**		**FDI**
opposition			
	**OP**		
	**ABP**		
	**FPB**		
	FPL		
	Add pol		

Add Pol: adductor pollicis; ADM: abductor digiti minimi; APB: abductor pollicis brevis; APL: abductor pollicis longus; ECR: extensor carpi radialis; ECU: extensor carpi ulnaris; EDC: extensor digitorum communis; EDM: extensor digiti minimi; EIP: extensor indicis propius; EPB: extensor pollicis brevis; EPL: extensor pollicis longus; FCR: flexor carpi radialis; FCU: flexor carpi ulnaris; FDI: first dorsal interosseous; FDP: flexor digitorum profundus; FDS: flexor digitorum superficialis; FPB: flexor pollicis brevis; FPL: flexor pollicis longus; OP: opponens pollicis.

**Table 2:** Muscles of lower extremity; primary muscles are in bold.

**Hip**				
	Flexion		Extension	
		**iliopsoas**		**Gluteus maximus**
		rectus femoris		hamstrings
		sartorius		
	Adduction		Abduction	
		pectineus		**Gluteus maximus**
		Adductor longus		Gluteus medius
		Adductor magnus		Gluteus minimus
		Adductor brevis		Tensor fascia lata
		gracilis		Obturator internus
				
**Knee**				
	Flexion		Extension	
		**hamstrings**		**quadriceps**
		popliteus		
		gastrocnemius		
		gracilis		
		sartorius		
**Ankle**				
	Dorsiflexion		Plantar Flexion	
		**Tibialis anterior**		**Gastrocnemius**
		EDL		**Soleus**
		EHL		plantaris
		Peroneus tertius		FHL
				FDL
	Inversion		Eversion	
		**Tibialis posterior**		fibularis/peroneus longus
		Tibialis anterior		fibularis/peroneus brevis
		FHL		fibularis/peroneus tertius
**Hallucis**				
	Flexion		Extension	
		**FHL**		**EHL**
				EHB
**Toes**				
	Flexion		Extension	
		FDL		EDL
		FDB		
		Quadratus plantae		

EDL: extensor digitorum longus; EHB: extensor hallucis brevis; EHL: extensor hallucis longus; FDB: flexor digitorum brevis; FDL: flexor digitorum longus; FHL: flexor hallucis longus.
